# Hypnotic suggestions cognitively penetrate tactile perception through top-down modulation of semantic contents

**DOI:** 10.1038/s41598-023-33108-z

**Published:** 2023-04-21

**Authors:** Marius Markmann, Melanie Lenz, Oliver Höffken, Agnė Steponavičiūtė, Martin Brüne, Martin Tegenthoff, Hubert R. Dinse, Albert Newen

**Affiliations:** 1 Department of Neurology, BG-Universitätsklinikum Bergmannsheil, Ruhr-University Bochum, Bochum, Germany; 2 Faculty of Social Sciences and Humanities, Klaipėda University, Klaipeda, Lithuania; 3 Department of Psychiatry, Psychotherapy and Preventive Medicine, LWL Universitätsklinikum Bochum, Ruhr-University Bochum, Bochum, Germany; 4 Institute of Philosophy II, Ruhr University Bochum, Bochum, Germany

**Keywords:** Neuroscience, Psychology, Medical research, Neurology

## Abstract

Perception is subject to ongoing alterations by learning and top-down influences. Although abundant studies have shown modulation of perception by attention, motivation, content and context, there is an unresolved controversy whether these examples provide true evidence that perception is penetrable by cognition. Here we show that tactile perception assessed as spatial discrimination can be instantaneously and systematically altered merely by the semantic content during hypnotic suggestions. To study neurophysiological correlates, we recorded EEG and SEPs. We found that the suggestion “your index finger becomes bigger” led to improved tactile discrimination, while the suggestion “your index finger becomes smaller” led to impaired discrimination. A hypnosis without semantic suggestions had no effect but caused a reduction of phase-locking synchronization of the beta frequency band between medial frontal cortex and the finger representation in somatosensory cortex. Late SEP components (P80–N140 complex) implicated in attentional processes were altered by the semantic contents, but processing of afferent inputs in SI remained unaltered. These data provide evidence that the psychophysically observed modifiability of tactile perception by semantic contents is not simply due to altered perception-based judgments, but instead is a consequence of modified perceptual processes which change the perceptual experience.

## Introduction

Human and animal brains reorganize continuously throughout lifespan due to neuroplastic processes. As a result, perception and behavior are not fixed but undergo major changes on a time scale of years to minutes^1–6^. In addition, perception is subject to ongoing alterations through top-down influences. Although abundant studies show modulation of perception by attention, motivation, content and context, there is an unresolved controversy in how far these examples provide true evidence that perception itself is penetrable by cognition^7,8^. The notion of cognitive impenetrability^8^ claims that cognition and perception are independent modules where perception is “shielded” from top-down influences related to cognitive processes^8–10^. Firestone and Scholl^8^ made this view recently prominent again: “We have argued that there is a joint between perception and cognition to be carved by cognitive science, and that the nature of this joint is such that perception proceeds without any direct, unmediated influence from cognition”. And they summarize their radical claim: “(…), it will remain eminently plausible that there are no top-down effects of cognition on perception.” It has been argued that the many studies that claim showing top-down influences and thus alterability of perception do not clearly distinguish the influence of cognition on experience-based *judgments* from the influence on the perceptual *experience*^8^. This view requires a modification of the perceptual *experience* but not that of judgement as a convincing case for penetrability^7,11^. Since most studies addressing top-down influences on perception deal with the visual system, we decided to broaden this issue by investigating a different modality. We developed a particularly designed experimental protocol employing tactile spatial discrimination ability to make progress in the debate about cognitive penetrability of perceptual experience.

There are only a few studies addressing top-down modulation in the somatosensory system. For example, in a recent study, participants were verbally influenced and conditioned about the effect of an inert cream to improve tactile perception^12^. In a subsequent objectively assessed measurement of temporal discrimination threshold, participants showed improved tactile sensitivity and lower discrimination thresholds, while a control group showed no effects implying that verbal suggestion together with conditioning can alter temporal discrimination abilities^12,13^. Several years ago, it was shown that the spatial acuity on the arm was higher when the view of the arm was magnified indicating that the perceived size of a relevant skin portion affects its spatial discrimination abilities^14^.

To make a case for penetrability and to demonstrate that tactile perception is in fact most likely alterable by top-down influences, we developed a protocol without any physical intervention or placebo-treatment: the latter means that we exclude the typical features of a placebo-experiments, namely that a person is actually taking a pill and that the suggestion, e.g. a reduction of pain due to taking a pill, is identical to the measured effect, namely the level of pain. We actually did use suggestions but hypnotic suggestions without any physical treatment. Most importantly, the measured outcome, namely the objectively assessed threshold in two-point discrimination, had nothing to do with the content of the hypnotic suggestions, which was about finger size. In addition, two-point discrimination was not altered in the condition of hypnosis without suggestion. Only the hypnotic suggestions with opposing contents about finger size (large versus small) resulted in specific bidirectional changes of two-point discrimination. We therefore argue that it was most likely the content of these hypnotic suggestions that caused the changes of thresholds. Another advantage of this approach is that it does not involve any perceptual learning and possible long-term changes in perception and cognition. There is agreement that hypnosis can be effectively used in clinical treatment and rehabilitation^15–18^. Furthermore, as hypnotic suggestion can alter mental states, hypnosis is increasingly used as a tool in contemporary cognitive and neuroscience research^19,20^. Previous studies on the influence of hypnotic suggestions on a visual oddball paradigm have shown that a suggested visual blockade can decrease performance, as well as decrease strength of the correlated P3b signal^21^, a signal associated with frontal-parietal memory load^22^, but found no significant changes in earlier components. Another study, implementing a “tunnel vision” suggestion found that highly suggestible participants reported lower visibility of stimuli, but no change in task performance, indicating that the suggestion’s influence was limited to attendance^23^.

In our approach we measured tactile spatial discrimination thresholds^24,25^ under different hypnotic conditions. We used the hypnotic suggestion “imagine your index finger gets 5 times bigger” and the hypnotic suggestion “imagine your index finger gets 5 times smaller” to induce instantaneous bidirectional alterations of tactile spatial perception. We predicted that a suggested bigger finger was associated with an improved discrimination ability as indicated by lower discrimination thresholds. For the suggestion „smaller finger” we expected impaired discrimination as indicated by higher discrimination thresholds.

To investigate the underlying brain processes associated with the different types of hypnotic suggestions and the resulting changes in tactile perception, we recorded paired-pulse evoked somatosensory potentials (ppSEP) in the primary somatosensory cortex (SI). Paired-pulse suppression serves as a marker of cortical excitability, and changes of excitability are interpreted as an indication of changes of processing of afferent incoming sensory information^26–28^. We also analyzed late evoked potentials (late SEPs), which have been connected to higher order cognitive processes related to attention and expectation. In addition, to address systematic changes of more global, wide-spread activity related to top-down influences, we recorded resting state electroencephalography (EEG) with a particular emphasis on interaction between S1 and frontal cortical areas.

In this work we demonstrate psychophysically and neurophysiologically a crucial role for hypnotic suggestions in controlling tactile perception. We find that tactile perception is altered bidirectionally according to the semantic content of the hypnotic suggestions. These changes are paralleled by a reduction of synchronization between frontal and somatosensory areas as well as changes in late somatosensory evoked potentials, but no changes in local excitability in primary somatosensory cortex. These data suggest an online top-down influence on perceptual processes triggered solely by semantic contents.

## Results

### Hypnosis induced changes of tactile perception

**Figure 1:**
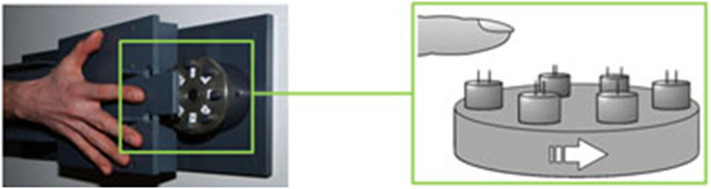
The two-point discrimination device used in the study. Seven different inter-pin distances (0.7–2.5 mm) and 1 control pin (0 mm) are presented to the index finger in random order.

We developed a protocol consisting of 4 experimental conditions (baseline, SUGG_0, SUGG_B, SUGG_S) within one day to explore how the semantic content of a hypnotic suggestion alters tactile perception and processing. A control condition without hypnosis served to find any impact of a neutral hypnotic condition on tactile discrimination abilities (baseline vs. SUGG_0). During the hypnotic condition SUGG_B participants were suggested the finger became bigger, while during the hypnotic condition SUGG_S participants were suggested the finger became smaller. In this way participants were enabled to experience opposing changes of tactile perception depending solely on the nature of the semantic content of the hypnotic suggestions. As a result, we expected enhanced tactile acuity under SUGG_B, but impaired acuity under SUGG_S. We measured tactile two-point discrimination (2ptD) thresholds in 24 participants. The device used allows for reliable assessment of discrimination thresholds and has been used previously in numerous studies ^24–26^ (Fig. 1). Four subjects had to be excluded because they were unable to perform the 2PD task reliably.

**Figure 2:**
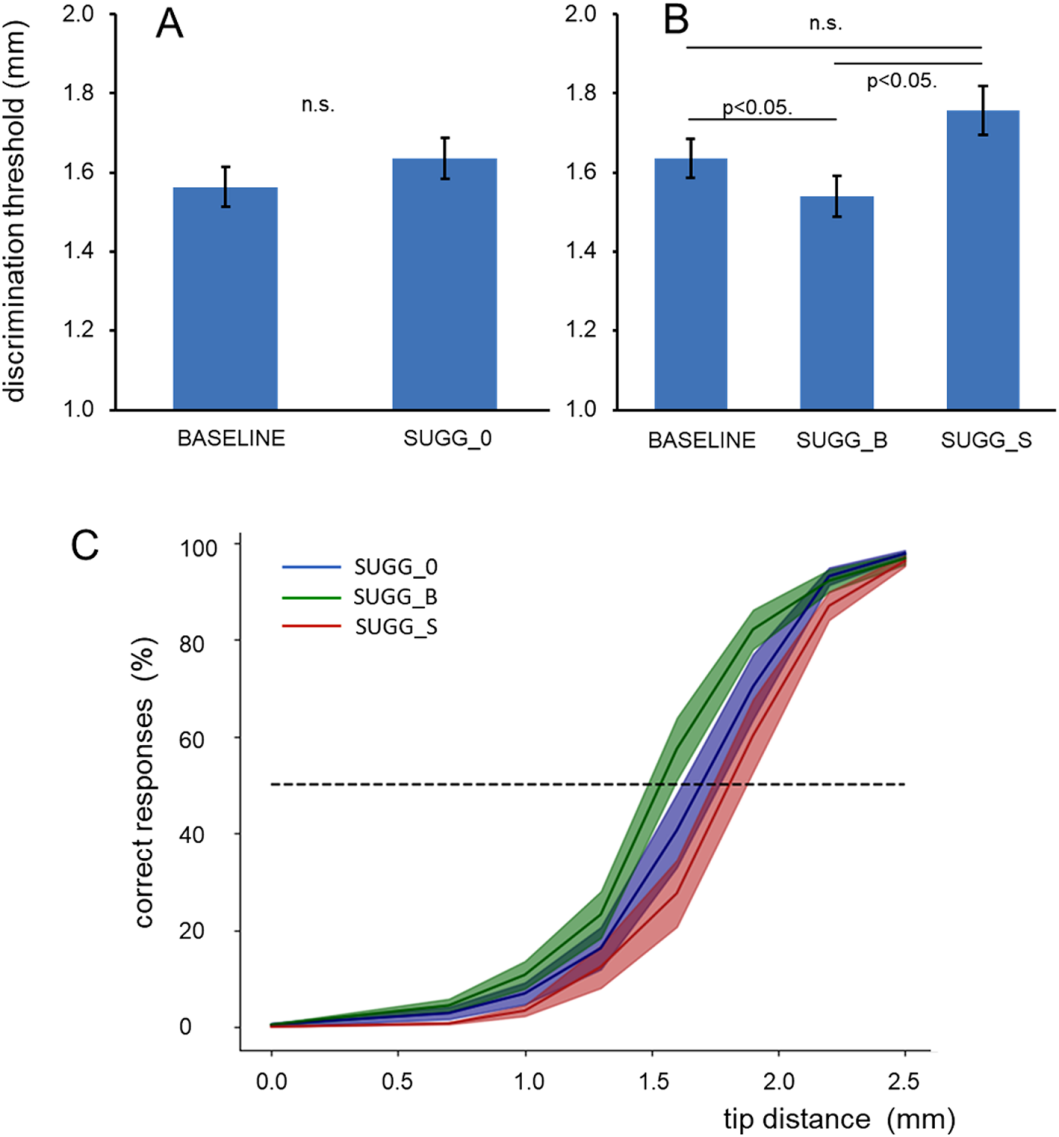
Effect of different hypnotic conditions on tactile discrimination threshold. Mean two-point discrimination thresholds ± SEM are shown in (**A**) for BASELINE and hypnosis without suggestion (SUGG_0), and in (**B**) for the conditions SUGG_0 (hypnosis without suggestion), hypnosis with the suggestion of a bigger index finger (SUGG_B), and hypnosis with the suggestion of a smaller index finger (SUGG_S). (**C**) Average psychometric curves ± SEM obtained from all participating subjects for the conditions hypnosis without suggestion (SUGG_0), hypnosis with the suggestion of a bigger index finger (SUGG_B), and hypnosis with the suggestion of a smaller index finger (SUGG_S).

Spatial discrimination thresholds obtained during the control condition baseline (mean 1.56, SEM 0.045) did not differ from threshold measured during a neutral hypnotic condition SUGG_0 (mean 1.63, SEM 0.050; rmANOVA F(1,19) = 2.403, *p* = 0.138, partial η^2^ = 0.112; N = 20) (see Fig. 2A). This finding is important as it shows that tactile acuity can be reliably assessed under hypnosis. In a next step we compared the 3 hypnotic conditions SUGG_0, SUGG_B and SUGG_S, which differed significantly (F(1.271,24.145) = 7.370, *p* = 0.002, partial η^2^ = 0.279). According to pair-wise post-hoc comparison (Bonferroni-corrected) thresholds obtained during SUGG_B were significantly lower as compared to SUGG_0 (*p* = 0.036) and SUGG_S (*p* = 0.025), while thresholds measured during SUGG_S were not significantly different compared to SUGG_0 (*p* = 0.121) (Fig. 2B). These results are illustrated as averaged psychometric curves in Fig. 2C. Improvement is shown by a shift of the curve to the left towards smaller separations, while impairment is indicated by a shift to the right towards larger separations.

**Figure 3:**
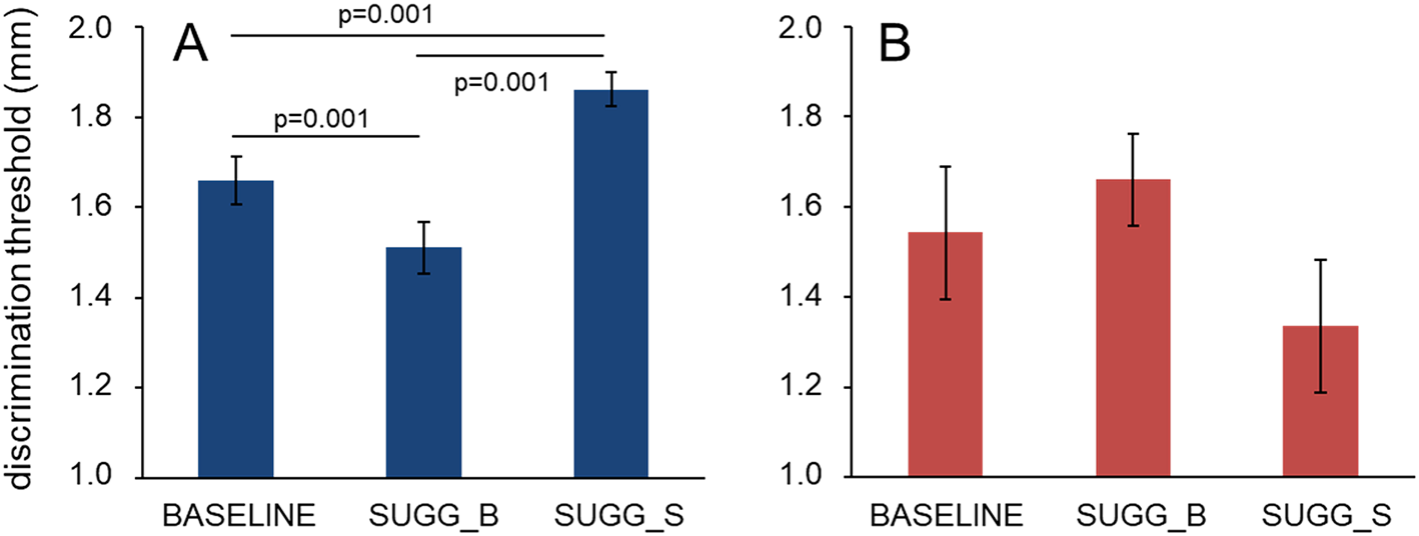
Average two-point discrimination thresholds ± SEM for responders (**A**) and non-responders (**B**). Responders showed decreased thresholds in SUGG_B and increased thresholds in SUGG_S, compared to SUGG_0. SUGG_0 = hypnosis without suggestion, SUGG_B = hypnosis with the suggestion of a bigger index finger, SUGG_S = hypnosis with the suggestion of a smaller index finger.

Since we already observed one clear behavioral effect of the hypnotic suggestion on the discrimination threshold, we decided to have a closer look on the behavior of the individual with the aim of investigating the underlying mechanism of this effect: is this an influence on the perceptual processes or just on the judgment based on it? A closer analysis revealed that only 16 out of 20 participants showed changes of tactile discrimination consistent with the hypothesis that the different semantic content embedded in the hypnotic suggestions leads to improved and impaired discrimination performance (Fig. 3A), namely improved discrimination via decreased discrimination threshold triggered by SUGG_B and the opposite for SUGG_S. One subject showed identical thresholds for both conditions containing a suggestion. However, because this participant otherwise resembled the response behavior seen in the group of responders, and particularly did not show the response seen in the non-responders, we did not exclude him from further analysis. The remaining 4 participants showed an opposite behavior in relation to the opposing suggestions and were therefore denoted as non-responder (Fig. 3B). In fact, in all 4 participants thresholds in the condition SUGG_B were higher than those observed in condition SUGG_S. Table 1 lists the individual discrimination thresholds in mm for the group of responders and the group of non-responders for all three conditions.

**Table 1:** Discrimination thresholds (mm) of all participants for three hypnotic conditions.

SUGG_0	SUGG_B	SUGG_S
Responder		
1.90	1.68	2.02
1.33	1.23	1.83
1.84	1.60	1.79
1.66	1.65	2.02
1.52	1.12	1.64
1.91	1.79	1.99
1.45	1.29	1.64
2.01	1.92	1.92
1.90	1.64	2.05
1.34	1.16	1.71
1.71	1.52	1.97
1.49	1.37	1.89
1.64	1.45	1.68
1.60	1.49	1.75
1.60	1.45	2.02
1.74	1.73	1.83
Non-responder		
1.56	1.64	1.07
1.95	1.95	1.75
1.33	1.49	1.22
1.33	1.56	1.30

Separate analysis (rmANOVA) for the group of responders confirmed first that there were no differences between baseline (mean 1.57, SEM 0.046) and neutral hypnosis (mean 1.65, SEM 0.052; F(1,15) = 2.660, *p* = 0.124; partial η^2^ = 0.151; N = 16).). Furthermore, as predicted, the three hypnotic conditions differed significantly (SUGG_0, SUGG_B, SUGGS; F(2,30) = 38.216, *p* = 0.001; partial η^2^ = 0.718) (Fig. 3A). According to pairwise post-hoc comparison (Bonferroni-corrected) thresholds of all three conditions differed significantly with *p* = 0.001. Because of the small sample size (N = 4), no statistics were calculated for the group of non-responders. These data show that the semantic content embedded in a hypnotic suggestion is able to evoke content-specific bidirectional alterations in tactile acuity.

### Effects of hypnosis on local and global neural processing

In search of neural underpinnings of the psychophysical demonstration that hypnosis within brief periods of time significantly alters tactile perception, we measured paired-pulse suppression as a marker of excitability of primary somatosensory cortex (SI) by means of SEP recordings following paired median nerve stimulation. Additionally, we investigated late SEP components (P45, N60, P80, N140) and peak-to-peak amplitudes (P45–N60, P100–N140) from single-pulse SEP in order to evaluate changes in higher perceptual brain regions like SII.

Functional cortical connectivity was assessed by recording ongoing EEG. While SI excitability can be interpreted as a marker of local processing, functional connectivity data serve as a measure of global interaction. For all neurophysiological measures, only data from responding participants were used to obtain potential neural correlates related to a consistent psychophysical response behavior.

### Effects on paired-pulse SEP

**Figure 4:**
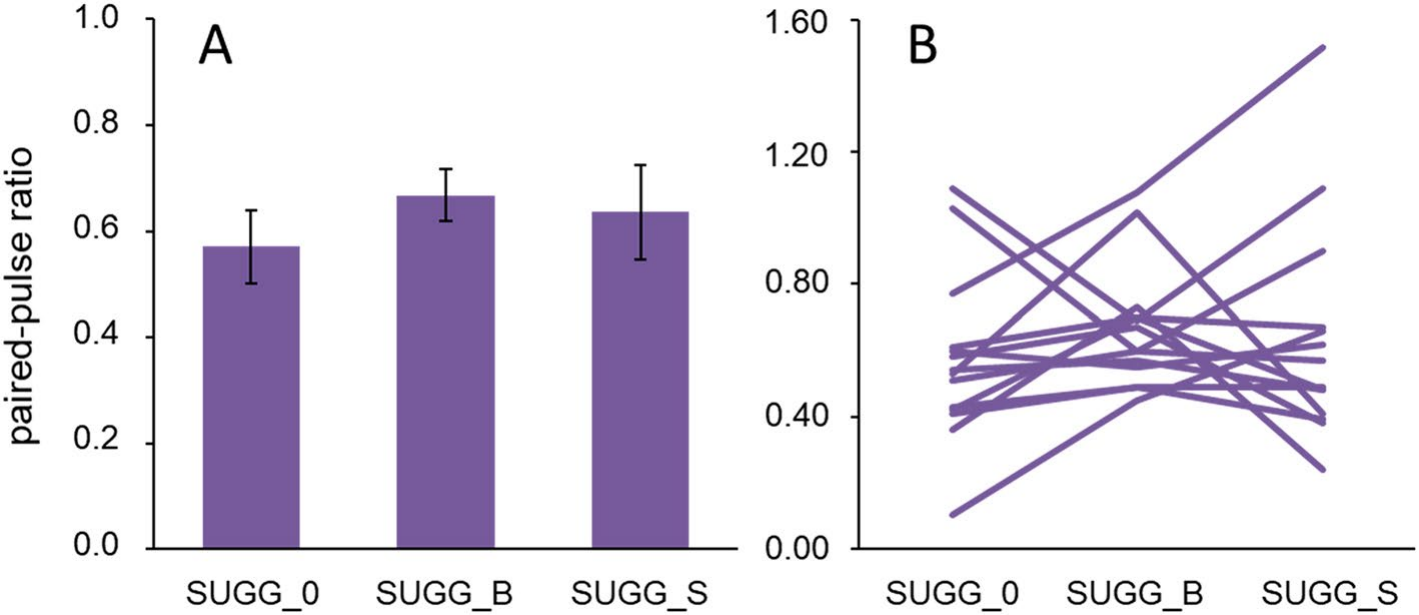
Effect of different hypnotic conditions on paired-pulse ratios. (**A**) Mean paired-pulse ratios are plotted for hypnosis without suggestion (SUGG_0), hypnosis with the suggestion of a bigger index finger (SUGG_B), and hypnosis with the suggestion of a smaller index finger (SUGG_S). (**B**) Individual paired-pulse ratios.

Analysis (rmANOVA) of paired-pulse ratios revealed that there were no differences between baseline (mean 0.632, SEM 0.038) and neutral hypnosis (mean 0.57, SEM 0.068; F(1,13) = 1.254, *p* = 0.283; partial η^2^ = 0.088; N = 14). This finding indicates that a neutral hypnotic condition does not affect paired-pulse suppression in SI. To clarify in how far semantic contents have an influence on paired-pulse suppression, we compared the 3 hypnotic conditions SUGG_0, SUGG_B and SUGG_S, which revealed that there were also no differences between conditions (F(2,26) = 0.848, *p* = 0.440; partial η^2^ = 0.061; N = 14) (Fig. 4A).

**Figure 5:**
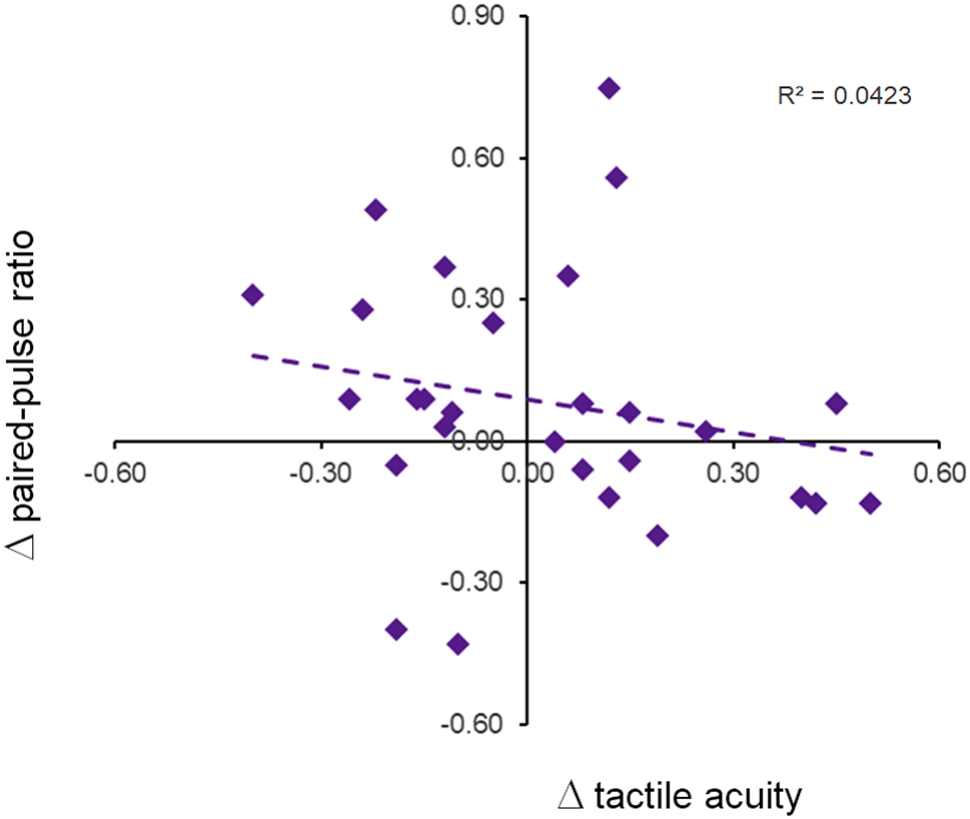
Correlation between changes of tactile discrimination and changes of paired-pulse ratios. Pearson correlation analysis between the differences in tactile acuity evoked by the semantic content of the hypnotic suggestion and associated differences of paired-pulse suppression. *R*^2^ = coefficient of determination.

We substantiated this observation by analyzing the individual time course of the paired-pulse data. While psychophysically all participants responded in a consistent way with the outlined hypothesis about opposing changes in acuity (Fig. 3A), the time course of paired-pulse suppression revealed a substantial inconsistency (Fig. 4B). We therefore further analyzed whether there is any relationship between the differences in tactile acuity evoked by the semantic content of the hypnotic suggestion and associated differences of paired-pulse suppression. A linear Pearson correlation analysis (r = − 0.206, *p* = 0.294, n = 28, explained variance 4.2%) revealed a clear lack of relationship (Fig. 5). These data imply that the sematic content can alter tactile acuity (Fig. 3A), but has no effect on paired-pulse suppression, which indicates that hypnotic suggestions have no influence on SI processing.

### Effects on late single-pulse SEP responses

**Figure 6:**
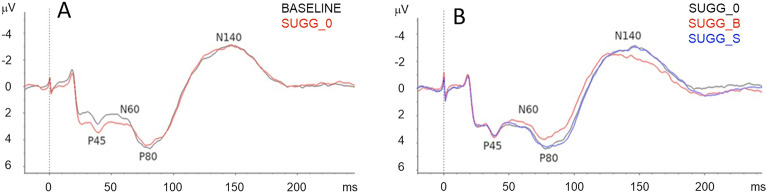
Grand-average waveforms. Recordings of the late SEPs in the Baseline and SUGG_0 condition (**A**) and in the three hypnotic conditions (**B**). Late SEP response peaks P45, N60, P80 and N140 are marked.

Grand averages of SEPs recorded after single-pulse median nerve stimulation for baseline and hypnosis (SUGG_0) conditions are shown in Fig. 6A, and for the three hypnotic conditions SUGG_0, SUGG_B, SUGG_S in Fig. 6B. The recordings show a typical waveform with the late response peaks P45 (41–53 ms), N60 (45–67 ms), P80 (71–95 ms) and N140 (125–164 ms).

**Figure 7:**
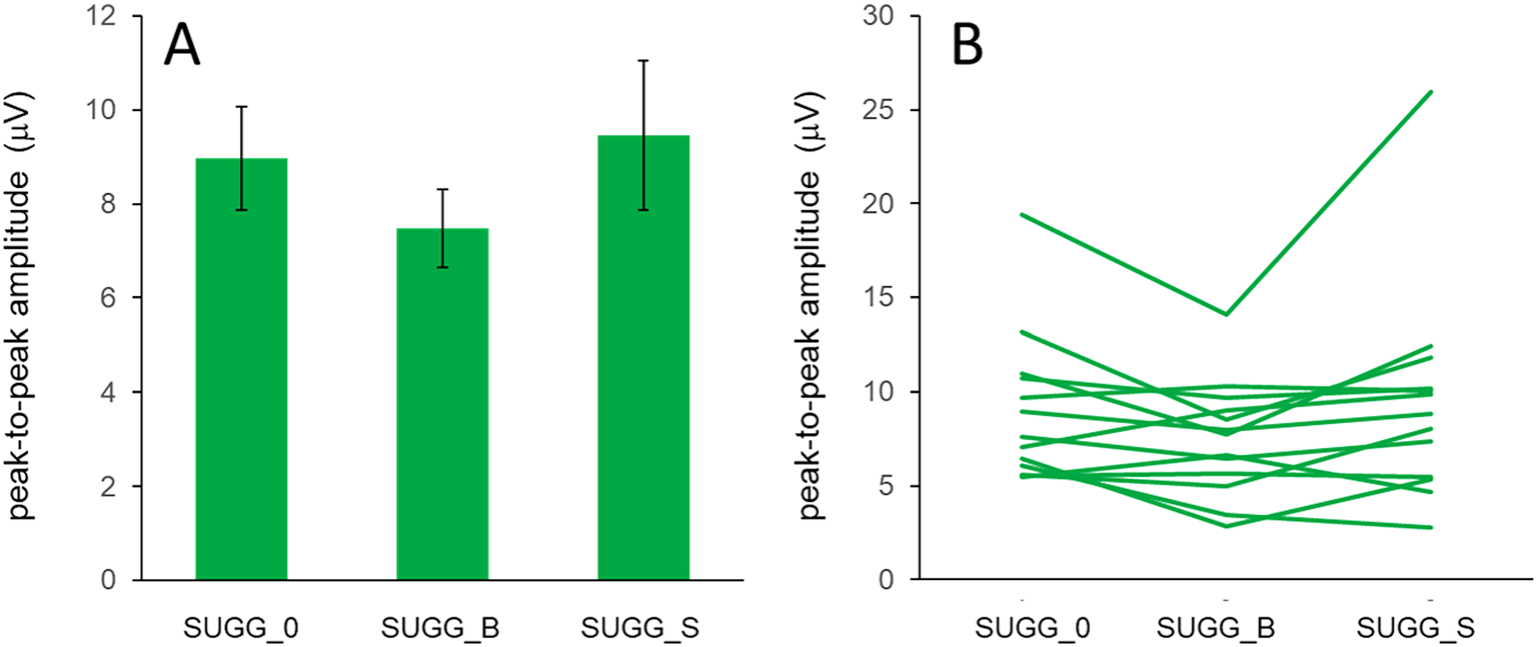
P80–N140 peak-to-peak amplitudes for different hypnotic suggestions. (**A**) Mean P80–N140 peak-to-peak amplitudes ± SEM are plotted for hypnosis without suggestion (SUGG_0), hypnosis with the suggestion of a bigger index finger (SUGG_B), and hypnosis with the suggestion of a smaller index finger (SUGG_S). RmANOVA revealed a significant effect of suggestions compared to hypnosis alone. (**B**) Individual P80–N140 amplitudes.

Analysis (rmANOVA) of individual peak-to-peak amplitudes revealed no significant differences between baseline and hypnosis without suggestion (SUGG_0), for none of the analysed peak amplitudes. In contrast, rmANOVA revealed a weak, though significant effect of suggestions compared to hypnosis alone (F(2,24) = 3.512, *p* = 0.046; N = 13) for the peak-to-peak amplitudes of the P80–N140 components (Fig. 6B); although post-hoc T-tests failed to reach significance (SUGG_B vs. SUGG_0, *p* = 0.106; SUGG_S vs. SUGG_0, *p* = 1.000; Fig. 7A). Individual P80–N140 amplitudes (Fig. 7B) revealed a substantial variability between subjects and conditions, explaining the lack of a significant influence of the hypnotic conditions.

### Effects of hypnosis and suggestions on neural network synchronization

**Figure 8:**
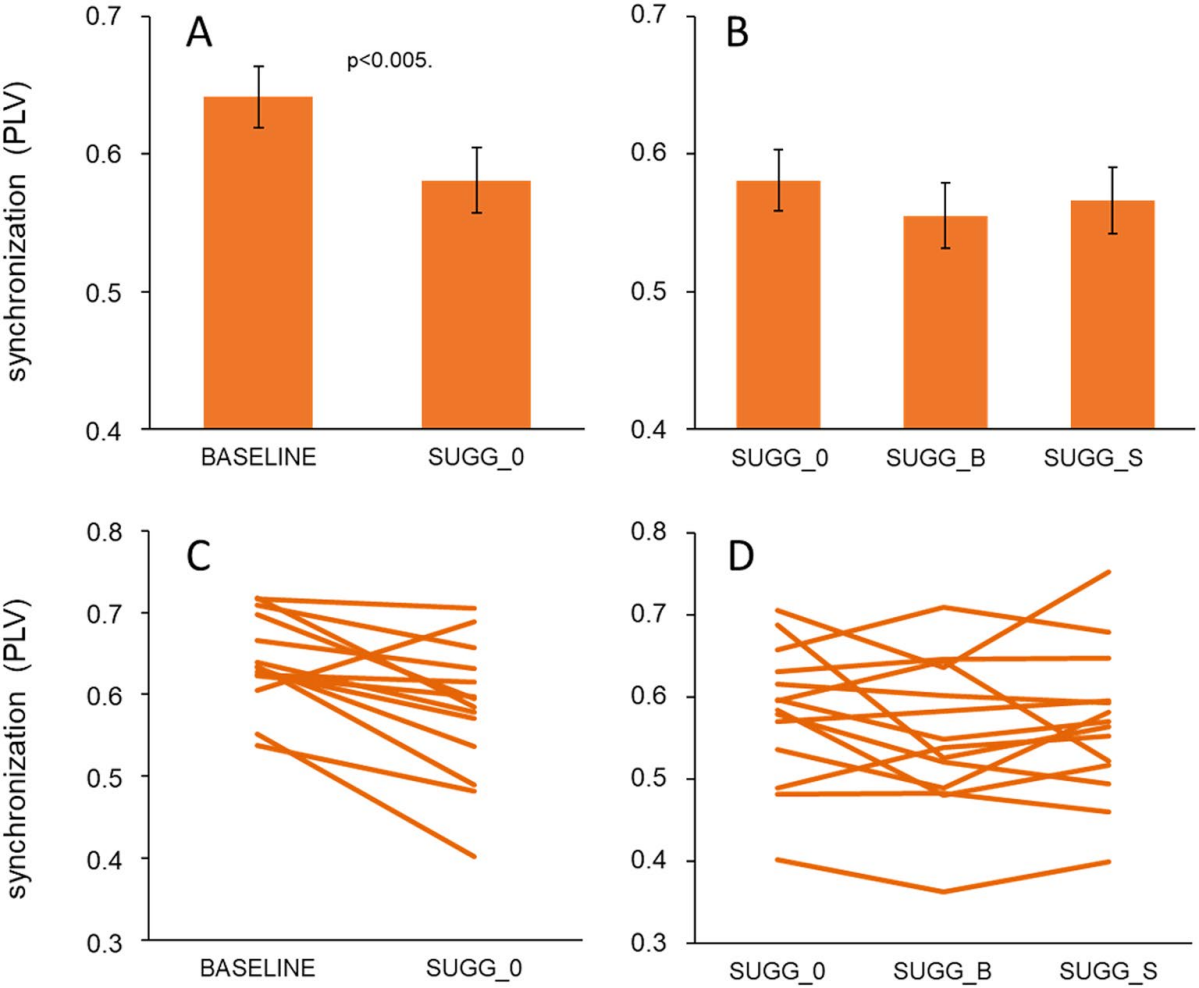
Resting-state functional connectivity for different hypnotic conditions. Phase-locking synchronization (PLV) ratios of the EEG beta (13–30 Hz) frequency band between left and right hemispheric medial frontal cortex (MFC) and the finger representation (CP3, CP4) in somatosensory cortex (SI). Mean synchronization after hypnosis without suggestion (SUGG_0) and baseline (**A**), and suggestions SUGG_0, SUGG_B and SUGG_S (**B**). (**C**, **D**) Individual changes of the PLV values.

As a next step we analyzed in how far resting-state functional connectivity was affected by the hypnotic conditions. We first analyzed phase-locking synchronization (PLV) of the EEG beta (13–30 Hz) frequency band between left and right hemispheric medial frontal cortex (MFC) and the finger representation (CP3, CP4) in somatosensory cortex (SI). To this aim, we pooled PLV between electrodes AF7_CP3, AF7_CP4, AF3_CP3, AF3_CP4, AFz_CP3, AFz_CP4, CP3_AF8, CP3_AF4, CP4_AF8 and CP4_AF4. From the 24 participants, 10 had to be excluded from analyses due to poor data quality. Analysis (rmANOVA) of the pooled PLV data revealed significant differences between baseline (mean 0.64, SEM 0.015) and the neutral hypnotic condition (mean 0.58, SEM 0.022; F(1,13) = 12.879, *p* = 0.003; partial η^2^ = 0.498; N = 14; Fig. 8A). This observation demonstrates that during hypnosis the synchronization between MFC and somatosensory cortex was reduced. However, further analysis showed that the semantic contents (SUGG_0, SUGG_B and SUGG_S) had no additional effect on PLV (F(2,26) = 1.480, *p* = 0.246; partial η^2^ = 0.246; N = 14; Fig. 8B).

Analysis of the individual changes of the PLV values showed that the decrease of synchronicity occurred very consistently in 13 out of 14 subjects analyzed (Fig. 8C). In contrast, the changes during the conditions SUGG_B and SUGG_S were much more variable (Fig. 8D).

Finally, we compared PLV in the alpha band for the connectivity between MFC and SI, which had revealed significant differences. There were no significant differences for any of the conditions tested except a weak trend for baseline vs SUGG_0: F(1,13) = 3.475, *p* = 0.085; partial h^2^ = 0.211; N = 14; SUGG_0, SUGG_B and SUGG_S: F(2,26) = 0.139, *p* = 0.871; partial h^2^ = 0.011; N = 14).

## Discussion

Our study demonstrates that the semantic content about finger size embedded in a hypnotic suggestion systematically alters the tactile perception as measured by spatial discrimination thresholds. We found that in 16 of 20 subjects, the suggestion “your index finger becomes 5 times bigger” lead to better tactile acuity, while the suggestion “your index finger becomes 5 times smaller” lead to impaired acuity. We focused on this group to investigate aspects of the neural basis of this effect. A neutral hypnosis without semantic suggestions had no effect on thresholds but caused a reduction of phase-locking synchronization (PLV) of the beta (13–30 Hz) frequency band between left and right hemispheric medial frontal cortex (MFC) and the finger representation (CP3, CP4) in somatosensory cortex (SI), which remained reduced across all hypnotic conditions. Late SEP components (P80–N140 complex) involved in attentional processes were altered by the semantic contents, but local processing of afferent inputs in SI as assessed by paired-pulse suppression indicative of excitability changes was not affected by any hypnotic condition.

Our data show that tactile perception is subject to specific modulation dependent on the semantic content transmitted through a hypnotic suggestion. The data further imply that despite major alterations in tactile acuity processing of the afferent input signals in SI is not similarly altered. Instead, the late P80–N140 complex was affected by the semantic content, indicating the involvement of attentional processing that further allows top-down influences to modulate the perception of the physical stimuli. In addition, we observed an uncoupling of SI processing from frontal areas that was evoked by hypnotic states but not further modulated by semantic contents. This might serve an unlocking stimulus processing from frontal cortical areas thereby facilitating an interpretation of the stimuli different from their physical appearance. Our findings are in line with other studies exploring states evoked by hypnosis or placebo-conditions showing comparable changes in connectivity^29,30^ or late SEP components^12^, although we did not find effects in paired-pulse behaviour^13^. However, our study differed in a number of methodological details. First, we compared the conditions under hypnotic suggestions (SUGG_B, SUGG_S) to a control condition where subjects were in hypnotic trance, but without an explicit suggestion (SUGG_0). More importantly, Perri et al.^31^ used a suggestion directly implicating the existence of a decreased tactile perception. In our study, we wanted to rule out the possibility that a direct suggestion like “your performance gets better or worse” might influence decision making instead of perception itself. Thus, we decided for more indirect suggestions like “your finger gets bigger” and “your finger gets smaller”.

Interestingly, four out of 20 subjects showed opposing effects as compared to the majority of the participants. While for the suggestion “the finger becomes smaller” 16 participants showed a decline in tactile acuity, the non-responders showed improved acuity. It is possible that these subjects misunderstood or misinterpreted the semantic content, or that simply the hypnosis failed to work appropriately. Independent of the reasons, to not confound the results of the responder, we decided to exclude these participants from further analyses.

Our data demonstrate that the frequency of the simple verbal report of the participants saying “1” or “2” when sensing a single touch or the touch of two needles was significantly altered under hypnotic suggestion, but not under a neutral hypnotic condition. Without any changes in the physical appearance of the stimuli used, the suggestion ‘imagine your finger gets bigger’ led to more reports of “2” indicating participants perceived a given separation between 2 stimuli as wider, while the suggestion “imagine your finger gets smaller” decreased the number of reports of “2”, indicating they perceived a given separation as less wide. This bidirectional response pattern points to an altered discrimination ability, where the semantic content “bigger” and “smaller” resulted in improved or impaired discrimination.

Do these data qualify as support for the notion of cognitive penetrability solely by the semantic content? Given the time course of the experiments, we can exclude that the effects are due to any kind of perceptual learning, since the semantic suggestion under hypnosis modified the discriminatory ability instantaneously. In our view, the only remaining explanation requires the assumption that the perceptual experience of the participants had be modified. This view is supported by a number of neurophysiological effects observed. During all hypnotic conditions, we observed a reduction of phase-locking synchronization (PLV) between left and right hemispheric medial frontal cortex (MFC) and the finger representation (CP3, CP4) in somatosensory cortex (SI).

In the condition of hypnosis without suggestion, we interpret this as a decrease of cognitively driven predominantly inhibitory processes, which channel and enable the normal interpretation of sensory inputs. This reduction of top-down influences has two effects: first, if there is no additional cognitive input coming into play, this results in an enhancement of bottom-up sensory processes due to lack of any top-down attentional processes—we understand attention as biased competition which may happen on all processing levels as developed in Marchi^32^. Second, if there is additional cognitive input delivered, typically by a hypnotic suggestion, then these new top-down influences are especially relevant, i.e. the content of this suggestion modifies the interpretation of the sensory data, which can be understood as re-establishing one specific top-down attentional focus.

The first effect is proven by many hypnosis studies, e.g. by studies with neutral hypnosis (without suggestions) the Stroop task performance becomes worse, with significant increases in reaction times or deteriorating accuracy rates^33–37^. These findings robustly demonstrate that hypnotic induction without any specific instructions inhibits top-down control processes thereby blocking all focused top-down attention, giving way for automatic bottom-up processes (for a detailed description see^38^, p. 103). These effects of hypnosis are also supported by electrophysiological studies^33^, functional neuroimaging^39^ studies, as well as behavioral tests assessing frontal functions such as verbal fluency^35,40^ (cf. also^41^) and a study on processing intentional actions of others^38^. The second effect,  i.e. the specific relevance of hypnotic suggestions, is support by many studies showing effects on cognitive phenomena and underlying neural processing in the case of hypnotic suggestions (for a review see^20^).

In addition to changes in connectivity, for the conditions containing semantic suggestions we observed a modification of late SEP components (P80–N140 complex), which have been implicated in attentional processes. These findings provide further support for the notion of top-down modulation of an otherwise unaltered sensory input during hypnotic suggestions. Interestingly, paired-pulse behavior reflecting intracortical inhibitory processes in SI remained unchanged under all hypnotic conditions. This observation is in line with the fact that during all conditions tested the afferent input remained identical. We therefore suggest that the altered perceptual experience was mediated by top-down influences. Possible signatures of a top-down modulation are the changes in late SEP components and the decoupling of SI from medial frontal cortex. Deeper insight into the mechanisms allowing for this massive impact of semantic content on perceptual experience might come from future hypnosis studies involving fMRI measurements.

It should be mentioned that all analyses of synchronization were performed on the electrode level, without the use of algorithms like LORETA, sLORETA and others. This was mostly due to technical limitations. In the present study, we only used a 32 electrode system, which made it possible to minimize the duration of the experiment which was one important aspect in the rather longish experiment, but hindered us from doing source level analysis. Classical source localization algorithms work best in cases where the data was recorded using EEG systems with 128 to 512 electrodes^42^; this can be improved in future research ideally in combination with fMRI measurements.

In our study, for estimating the hypnotisability of the participants, we used a protocol similar to the one by Shor and Orne^43^. However, we altered the method of report from using a self-report questionnaire to an evaluative judgement performed by the experienced hypnotist. Even though a self-report measure is advantageous for an analysis of the perceived internal state, relying on external judgements was both more time efficient and allowed for a better judgement of the hypnotisability in general. This is in line with recent discussion in hypnosis research, demonstrating a low internal consistency of hypnotisability scales (see^44^). As we were not concerned with the nature of the hypnotic experience for this experiment, external judgement was sufficient to validate general hypnotisability.

Recent work has questioned whether perception is penetrable by cognition. We used different semantic contents delivered during hypnotic suggestions in combination with a tactile spatial discrimination task to demonstrate that the semantic contents bidirectionally modifies tactile perception. This approach differs from others by not using magnification of the body parts tested or a physical intervention such as skin creams. Thus, we propose that the best explanation for our findings is a top-down effect on the perceptual experience by cognitive penetration mediated only by the semantic content of the hypnotic suggestion. The parallel changes in connectivity and late SEPs support this interpretation. Using semantic contents conveyed during hypnotic suggestions might in general be a powerful tool to interfere with perceptual experiences beyond the sense of touch.

## Methods

### Participants

We tested 24 right-handed participants aged 21–30 years (14 female, mean-age: 25.2 ± 2.7), left-handedness was both an exclusion criterion and checked on by means of the Edinburgh Handedness Inventory^45^.

Participants were invited in groups of 10–12 prior to the main experiment to test general hypnotisability. Hypnotisability was assessed by an experienced hypnotist, using a procedure similar to the Harvard hypnotisability scale^46^. Additionally, psychological health was assessed by the MINI-DIPS^47^ by a psychologist. Also, two-point-discrimination performance was tested in a short version of the experimental two-point-discrimination task in order to find out whether reliable thresholds can be measured in the respective subject. Eligible participants were invited for the main experiment. Informed consent was obtained from all subjects (no minors below 16 years of age involved).The study was approved by the Ethics Committee of the Ruhr-University of Bochum (16-5884-BR) and was conducted in accordance with the Declaration of Helsinki.

### Procedure

For the main experiment, participants were invited individually and were hypnotized and brought out of hypnosis before testing, to ensure fast hypnosis induction during testing. Participants were measured under four different conditions: 1. Without hypnosis and hypnotic suggestions (Baseline); 2. Under hypnosis, but without hypnotic suggestions (SUGG_0); 3. Under hypnosis, with the suggestion that the finger became 5 times bigger (SUGG_B); 4. Under hypnosis, with the suggestion that the finger became 5 times smaller (SUGG_S). The suggestions were given in German: “fünf mal größer” “fünf mal kleiner”.

Depending on the individual resting time, a few minutes separated the conditions.

Three different variables were measured under all 4 conditions: 1. Two-point-discrimination performance (2PD) 2. Resting-state EEG 3. Single- and paired-pulse evoked somatosensory potentials (SEPs).

**Figure 9:**
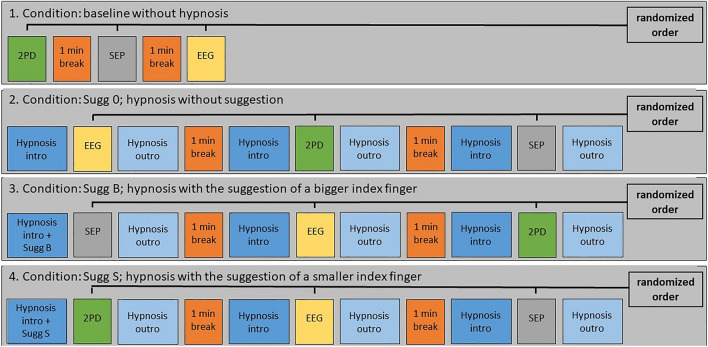
Study schedule. Experiments always started with the baseline condition, always followed by the SUGG_0 condition: hypnosis without suggestion. Afterwards, the SUGG_B and SUGG_S conditions were performed in a pseudo-randomized order. Also, all experimental tests (2PD, EEG, SEP) were performed in pseudo-randomized order in all conditions. *2PD* Two-point discrimination, *SEP* Somatosensory evoked potentials, *EEG* Resting-state electroencephalography.

The experiment always started with the baseline condition. After all three measurements were completed under baseline condition, hypnosis was re-induced by the hypnotist, and all tests were performed under hypnosis without suggestion (SUGG_0). In the 3rd and 4th condition, which were carried out in randomized order, a suggestion was added. At the beginning of the SUGG_B and SUGG_S sessions, the hypnotist briefly re-induced the hypnotic trance and gave the respective suggestive instructions (see the “Hypnosis” section for detailed description). See Fig. 9 for a schematic schedule of the study.

### Hypnosis

Participants were screened for eligibility by an experienced hypnotist, using a protocol similar to the Harvard Group Scale of Hypnotic Susceptibility (HGSHS^46^), comprising most of the items (e.g. olfactory hallucinations, “numb” limbs, eye catalepsy). In contrast to the HGSHS, we used no self-reporting scale in our study. Instead, the hypnotist rated the depth of the hypnotic state for each participant. Participants that were able to experience these hypnotic suggestions were considered eligible for further testing.

The first hypnotic induction in the study was done before the application of the EEG system, using a similar protocol to the hypnotic induction performed in the HGSHS. Subsequently the participants’ hypnoses were interrupted. Before measurements that required hypnosis with or without hypnotic suggestions (SUGG_0, SUGG_B and SUGG_S), participants were again given a brief hypnosis induction routine. For the SUGG_B-condition, hypnosis induction was followed by the suggestion “You now get a drug that has no side effects, but lets your index finger get five times bigger, nod if you can feel it”. For the SUGG_S-condition, hypnosis induction was followed by the suggestion “You now get a drug that has no side effects, but lets your index finger get five times smaller, nod if you can feel it”. After each participant nodded, the experiments were continued.

### Resting-state EEG

All EEG measurements were performed using an active 32 electrode system (actiCap, Brain Products GmbH, Wörthsee, Germany), connected to a 32-channel amplifier (BrainAMP MR, Brain Products GmbH, Wörthsee, Germany; bandpass-filter: 100–2000 Hz). All resting-state EEG measurements had a duration of 15 min while participants were seated in a comfortable chair with their eyes closed. Offline, eye-artifacts were removed from the EEG data using the VisionAnalyzer software (Version 2.1; Brain Products GmbH, Wörthsee, Germany). Further analyses were conducted using the Fieldtrip-Toolbox (Version 2.0190410; Donders Institute for Brain, Cognition and Behaviour, Radboud University, Netherlands) running on a Matlab R2019a (The Mathworks Inc., Natick (MA), USA) platform. The recorded data was downsampled to 250 Hz, and a bandpass filter (0.1–50 Hz) was applied. Trials were defined to be 2 s of length with 0.5 s overlap at the start and end of each trial. For bandwidth analysis we used a multitaper frequency transformation, exporting to the power domain, of 4 frequency bands, using a dpss taper (delta = [0.1–4 Hz], theta = [4–8 Hz], alpha = [8–13 Hz], beta = [13–30 Hz]). For connectivity analysis, we used the preprocessed data and performed a multi taper fast Fourier analysis, using a dpss taper, with Fourier data as an output, for the 4 frequency bands listed above. Connectivity analysis was performed on all frequency bands using the phase-locking value^48^.

### Single- and paired-pulse evoked somatosensory potentials (SEPs)

**Figure 10:**
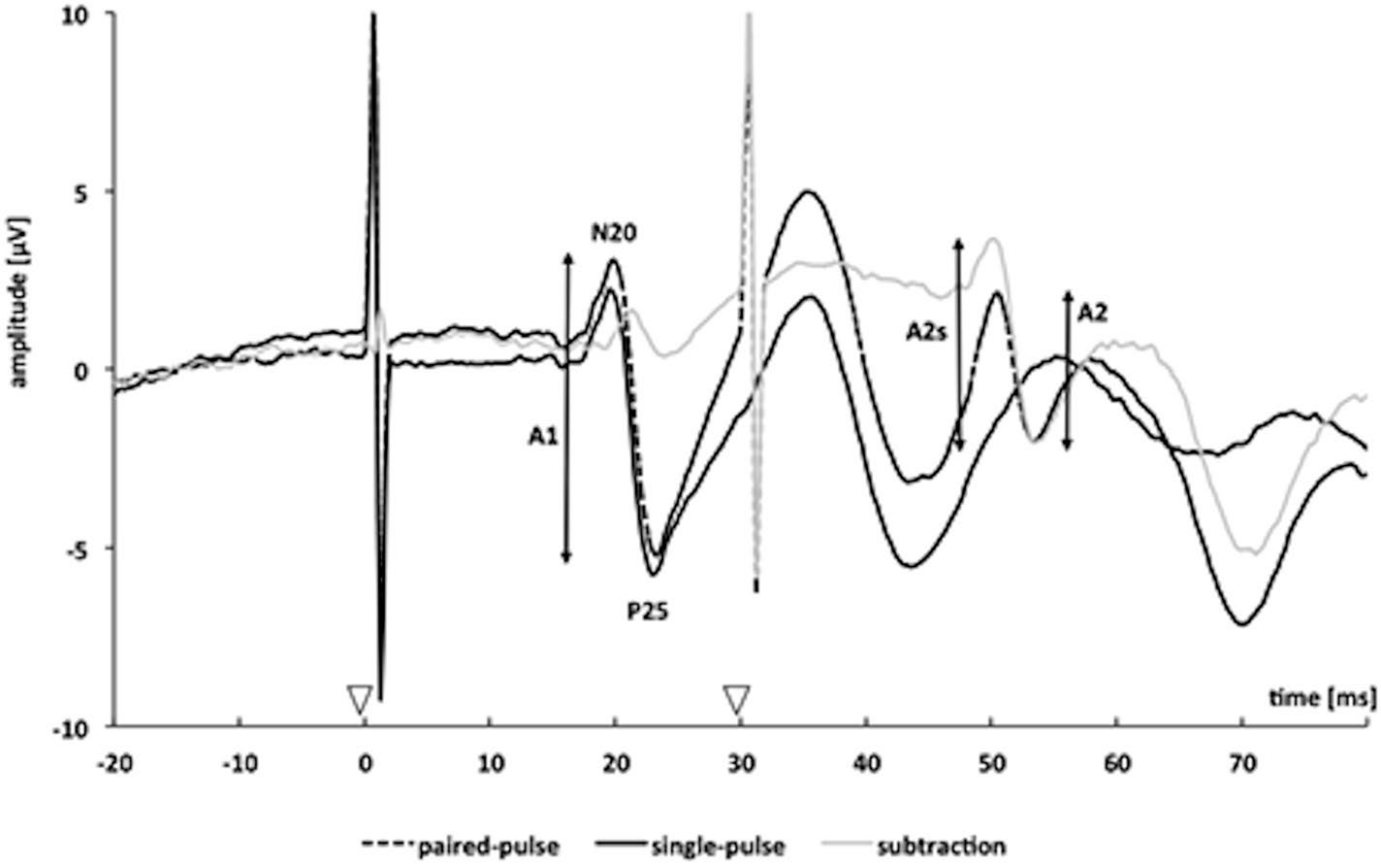
Representative paired-pulse somatosensory evoked potential of one subject. Somatosensory evoked potentials were measured over cortical CP3 or CP4 after single (continuous black trace) and paired-pulse stimulation with an interstimulus interval of 30 ms (continuous grey trace). The dotted black trace results by subtracting the single-pulse trace from the paired-pulse trace. The analyzed amplitudes of the first response (A1) and second response (A2) after paired-pulse stimulation are marked by vertical bars; amplitudes of the second response after subtracting the response to a single pulse are denoted as A2s. Onsets of the applied electrical stimuli are marked by arrowheads.

SEPs were measured following electrical stimulation of the median nerve. Before each measurement, the position of the block electrode was adjusted so that participants perceived a prickling sensation in the thumb, index and middle finger of the stimulated hand, stimulation intensity was adjusted to roughly double the perceptive threshold, so that there was a perceivable twitch in the thenar muscles. Participants were seated comfortably and were asked to close their eyes during the measurement. Cortical excitability was accessed using a paired-pulse protocol, consisting of paired electrical stimulation of the median nerve with an SOA (stimulus onset asynchrony) of 30 ms. Single pulse SEPs were recorded in order to be able to eliminate effects of superposition of the first and second paired-pulse stimuli (cf. Fig. 10). Thus, subjects received alternating single- and paired-pulse stimulation with both a pulse duration of 0.2 ms and 3 Hz repetitive rate. Both, single-pulse SEP (spSEP) and paired-pulse SEP (ppSEP) were applied 800 times. SEP were recorded using the same EEG setup as used for the resting state EEG (32-channel amplifier; BrainAMP MR, Brain Products, Wörthsee, Germany; bandpass-filter: 100–2000 Hz) and stored offline. SEP signals were analysed over the left SI using the CP3 electrode site with reference applied over the midfront (Fz) position, according to the international 10–20 system^49^.

Offline preprocessing was conducted separately for spSEP and ppSEP and involved segmentation of the signal in epochs of 20 ms before and 200 ms after stimulus onset, baseline correction and semi-automatic artefact rejection (muscle and eye-movement, amplitudes > 100 μV). SEP were then averaged over trials. Peak-to-peak amplitudes of the cortical N20–P25 SEP components were analyzed^26,50,51^. As shown in Fig. 10, after paired-pulse stimulation the response to the second pulse “rides” on the response to the first pulse, leading to a superimposition of both evoked potentials. Therefore, the amplitude of the response to the second pulse may misleadingly appear to be higher or lower. To assess “true” paired-pulse interaction, linear superposition effects had to be factored out by subtracting the response to the single-pulse stimulation from the paired-pulse stimulation trace. We analysed the second ppSEP amplitude after linear subtraction of the spSEP (A2s) and referred it to the first ppSEP amplitude before linear subtraction (A1). PPS was expressed as a ratio (A2s/A1) of the amplitudes of the second (A2s) and the first (A1) peak^26,50,51^ (Fig. 10).

### Late SEP responses

For the analysis of late SEP components, the EEG was band-pass filtered between 0.5 and 100 Hz (2nd order zero phase shift Butterworth filters) and segmented giving epochs of 320 ms (− 20 to 300) with the first 20 ms serving as the baseline. The segmented and baseline corrected trials were finally averaged, and the grand averages of SEP recorded in the four conditions were obtained. For statistical analyses, individual SEP Peak detection was also conducted and mean peak amplitudes were compared between conditions.

### Tactile spatial acuity assessment

Two-point discrimination (2ptD) thresholds were assessed on the tip of the index finger (D2) of both hands by using the method of constant stimuli^52–54^. This procedure corresponds to an improved version of the classical 2-point discrimination task^24,25^. In this version, the threshold does not correspond to the distinction between 1 tip versus 2 tips, but to the decision when 2 tips are sufficiently separated to be perceived as two. To this aim, the entire psychometric curves were computed and then used to determine the distance at which participants reported the sensation of two clearly separated tips. All subjects underwent one training session in order to familiarize themselves with the testing procedure in the first session. A custom-made device was used to assess the 2ptD thresholds at a fixed location on the skin of the fingertips by rapidly switching between stimuli (see Fig. 1). The stimuli consisted of 7 pairs of brass needles with individual spacing ranging from 0.7 to 2.5 mm in increments of 0.3 mm and a single needle as zero distance (control condition). Brass pins were 0.7 mm thick with blunt tips of approximately 200 μm diameter. Tactile stimuli were applied for approximately 1 s with application forces ranging between 150 and 200 mN. The subjects were instructed to place their finger on the support and to maintain this initial position of the finger throughout the experiment. The down movement was stopped at a fixed position above the pins. The index finger of the right hand was placed above a small hole through which the finger touched the tips of the pins at approximately the same indentations in each trial^52–54^. Subjects were not informed about the ratio of paired to single needles being 7:1. The participants had to report immediately after stimulus contact if they had the sensation of 1 or 2 needles being applied by reporting the percept of a single needle or any ambiguous stimulus as “1”, and the distinct percept of 2 needle tips as “2.” Emphasis was laid on answering “two” only when clearly perceiving two distinct points. When perceiving a bar, a bigger point or any unclear shape, participants were instructed to answer “one”. To obtain a stable baseline discrimination performance, each distance was tested eight times in pseudorandomized order in each condition, resulting in a total of 64 trials. The summed responses were plotted against distance as a psychometric function for absolute threshold, fitted by a binary logistic regression. Threshold was taken from the fit at the distance for which 50% correct responses were reached.

## Data Availability

Data can be made available upon reasonable request from the corresponding authors.
